# Effect of Cap-Lenticule Diameter Difference on the Visual Outcome and Higher-Order Aberrations in SMILE: 0.4 mm versus 1.0 mm

**DOI:** 10.1155/2017/8259546

**Published:** 2017-11-15

**Authors:** Banu Torun Acar, Suphi Acar

**Affiliations:** Bati Goz Hospital, Istanbul, Turkey

## Abstract

**Purpose:**

To evaluate the effect of cap-lenticule diameter difference (CLDD) on the visual outcome and higher-order aberrations (HOAs) of small-incision lenticule extraction (SMILE).

**Methods:**

A total of 132 patients who had bilateral SMILE for myopia or myopic astigmatism were included. The CLDD was 0.4 mm in 54 patients (group 1) and 1.0 mm in 78 patients (group 2). The refractive parameters, uncorrected (UDVA) and corrected distance visual acuity (CDVA), and HOAs were determined preoperatively and during six months follow-up.

**Results:**

Group 1 had better CDVA (in logMAR) compared to group 2 at day 1 (−0.07 ± 0.07 versus 0.04 ± 0.07, resp.; *p* < 0.001) and week 1 (−0.07 ± 0.07 versus –0.04 ± 0.07, resp.; *p* = 0.001). The visual acuity improved more in group 1 than in group 2. The UDVA (in logMAR) was 0.07 ± 0.07 and 0.29 ± 0.09 at day 1 (*p* < 0.001) and −0.08 ± 0.07 and −0.06 ± 0.06 at six months (*p* = 0.038) in group 1 and group 2, respectively. Group 1 was associated with significantly less induction of HOAs (0.24 ± 0.08 *μ*m and 0.32 ± 0.26 *μ*m, resp.; *p* = 0.002).

**Conclusions:**

In SMILE, 0.4 mm CLDD is associated with better visual outcome and less induction of HOAs than 1.0 mm. Narrow CLDD should be considered in SMILE to increase the visual acuity particularly in the early postoperative period.

## 1. Introduction

Myopia or myopic astigmatism is a benign refractive error with an increasing prevalence worldwide [1]. It can be corrected permanently by laser-assisted in situ keratomileusis or refractive lenticule extraction [2, 3]. Small-incision lenticule extraction (SMILE) is a novel technique first developed in 2008 [4]. Currently, SMILE and femtosecond laser-assisted laser in situ keratomileusis (LASIK) are the most popular refractive procedures performed by femtosecond laser to correct myopia and myopic astigmatism [5].

SMILE is performed by producing an intrastromal lenticule with the femtosecond laser and extracting it through a small incision. It is a minimal invasive corneal refractive surgery [6]. SMILE and femtosecond LASIK have comparable efficacy and safety profiles, as SMILE has the benefits of less induction of higher-order aberrations (HOAs), superior biomechanics, greater corneal sensitivity, and fewer dry eye symptoms [7–10]. The only disadvantage of SMILE is that visual acuity does not recover after SMILE as rapidly as in LASIK [11]. R. Shah and S. Shah reported that ReLEx SMILE and ReLEx FLEX have a slower visual recovery compared to LASIK operations but the effectiveness and safety are comparable [12]. Visual outcome is being improved by modifying the scanning trajectory of the femtosecond laser. Also, Vestergaard et al. and Kamiya et al. reported that visual recovery after lenticule extraction in SMILE is slower than after LASIK [13, 14].

Although visual acuity obtained by SMILE is comparable to LASIK in the long term, it may remain at a lower level in the early postoperative period. Various techniques including intraoperative cap repositioning, changing cap thickness, and lowering laser energy levels have been suggested to enhance visual recovery in the short term [15–18]. However, the effect of the difference between diameters of the cap and lenticule on early visual and refractive outcome has not been investigated so far. According to the professional use information of VisuMax Femtosecond Laser (Zeiss, Carl Zeiss Meditec, Jena, Germany), cap-lenticule diameter difference (CLDD) is suggested to be within 1.0–1.1 mm. Based on our clinical experience, we hypothesized that visual and refractive outcome in early postoperative period increases as CLDD during SMILE procedure decreases.

In this study, we aimed to evaluate the effect of CLDD on the visual outcome and HOAs in SMILE by comparing 0.4 mm versus 1.0 mm CLDD in a cohort of 132 patients with myopia or myopic astigmatism.

## 2. Patients and Methods

### 2.1. Study Population and Groups

This was a prospective comparative clinical study in which 132 consecutive patients who had bilateral SMILE in our clinic between March 2016 and December 2016 participated. The inclusion criteria were 18 years of age or older, diagnosis of myopia with a spherical equivalent of −1.00 to −10 diopters (D) or astigmatism of 0–4 D, corneal thickness > 500 *μ*m, stable refraction in the last two years, and normal corneal topography. Eyes with other ocular diseases, severe dry eye, progressive corneal degeneration, cataract, or uveitis were excluded from the study. The first 50 patients who had SMILE operation during study period were excluded due to learning curve. One of two CLDD parameters was utilized during SMILE, and patients were consecutively divided into two groups accordingly: CLDD was 0.4 mm in group 1 (*n* = 54 patients) and 1.0 mm in group 2 (*n* = 78 patients).

The study was conducted according to the Helsinki Declaration and approved by the Institutional Ethics Committee. All patients gave written informed consent before any study-related procedures.

### 2.2. SMILE Procedure

Regular corneal topographic pattern was confirmed by Sirius™ topography system (Costruzione Strumenti Oftalmici, Firenze, Italy) before the SMILE procedure. The residual thickness of the stromal bed was >250 *μ*m, and mesopic (4 lux) pupil diameter was ≤6.5 mm in all patients. SMILE was performed using the VisuMax femtosecond laser (Carl Zeiss Meditec, Jena, Germany) with a 500 kHz repetition rate as described previously [19]. The femtosecond laser with an energy of 130 nJ was used to create the four cleavage planes. The small incision, 2 mm, was located in the 120° position. The spot spacing and tracking spacing were 4.5 *μ*m for the cap and lenticule and 2.0 *μ*m for the side cut. The following parameters were used: cap thickness 120 *μ*m; cap diameter 6.9 mm and lenticule diameter 6.5 mm in group 1; cap diameter 7.5 mm and lenticule diameter 6.5 mm in group 2. All the procedures were performed by the same surgeon (BA).

Postoperatively, all patients received topical ofloxacin 0.3% and fluorometholone 0.1% ophthalmic suspension four times a day for two weeks, with the dose being reduced gradually thereafter. Preservative-free artificial teardrops were used for one month postoperatively.

### 2.3. Ocular Examination and Outcome Measures

Patients underwent a complete ophthalmic examination preoperatively and at day 1, week 1, and months 1, 3, and 6 postoperatively. Preoperative examinations included logMAR of UDVA and CDVA, manifest refraction, slit-lamp biomicroscopy, dilated fundoscopy examination, and corneal topography. CDVA was measured using a Snellen chart at 6 meters in a well-illuminated room. All examinations were performed by an experienced ophthalmic technician.

The refractive parameters—sphere and spherical equivalent—were determined preoperatively and at month 6 postoperatively. To evaluate visual outcome of SMILE, UDVA and CDVA were measured at day 1, week 1, and months 1, 3, and 6 postoperatively. The anterior corneal aberrations (HOAs, horizontal coma, vertical coma, and spherical aberrations) were measured over the 6.0 mm diameter central corneal zone by the Sirius R device (Costruzione Strumenti Oftalmici, Firenze, Italy) preoperatively and six months after surgery.

### 2.4. Statistical Analysis

Statistical analysis was performed using SPSS for Windows (Statistical Package for Social Sciences, ver. 16.0, SPSS Inc., Chicago, Illinois, USA) software. Study data were summarized using descriptive statistics (e.g., mean, standard deviation). For comparison of continuous variables of two groups, independent sample *t*-test was used. To evaluate the significance of intragroup change during study, paired *t*-test or repeated measures analysis of variance (ANOVA) was used for two or more than two measurements, respectively. Statistical level of significance was set to *p* < 0.05.

## 3. Results

A total of 108 eyes in group 1 and 156 eyes in group 2 were studied. There was no statistically significant difference between study groups in terms of age (29.25 ± 5.36 years versus 29.54 ± 5.03 years, resp.) and gender of patients (61% female versus 59% female, resp.) (Table 1).

### 3.1. Correction in Refractive Parameters

**Figure 1:**
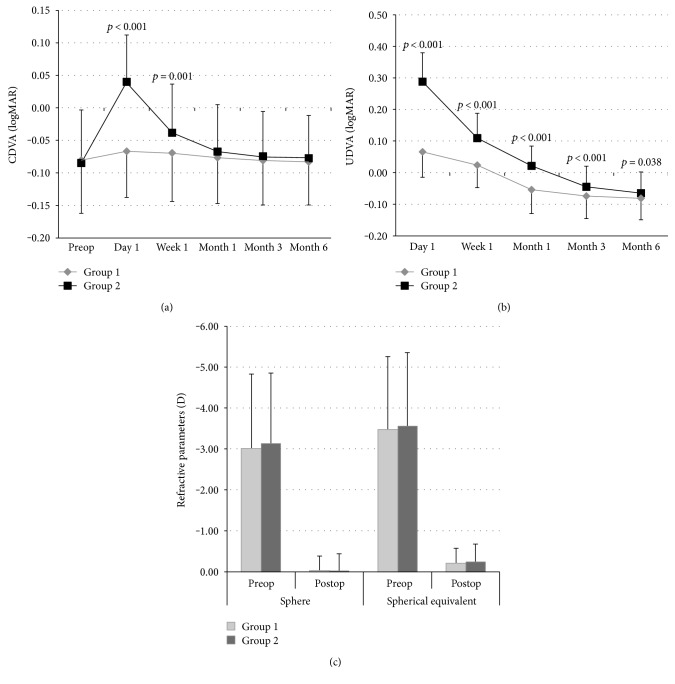
The mean and CDVA (a) and UDVA (b) in logMAR and sphere and spherical equivalent (c) in D of group 1 and group 2 during 6 months follow-up after SMILE procedure. The mean UDVA was significantly higher in all time points, as the mean CDVA was significantly higher in day 1 and month 1 postoperatively in group 2 than in group 1. SMILE provided statistically significant correction in both sphere (*p* < 0.001) and spherical equivalent (*p* < 0.001) in both groups. But both did not show significant difference between groups before and after the SMILE procedure (*p* = 0.582 and *p* = 0.943, resp.). CLDD: cap-lenticule diameter difference; CDVA: corrected distance visual acuity; UDVA: uncorrected distance visual acuity.

Postoperative assessment at month 6 showed that SMILE provided a statistically significant correction in both sphere (from −3.01 ± 1.81 D to −0.02 ± 0.36 D in group 1, *p* < 0.001; from −3.13 ± 1.72 D to −0.01 ± 0.42 D in group 2, *p* < 0.001) and spherical equivalent (from −3.47 ± 1.78 D to −0.20 ± 0.37 D in group 1, *p* < 0.001; from −3.55 ± 1.80 D to −0.24 ± 0.43 D in group 2, *p* < 0.001). However, the mean sphere and spherical equivalent of study groups were similar before and after the SMILE procedure (*p* = 0.582 and *p* = 0.943, resp.; Table 1, Figure 1).

### 3.2. Visual Acuity

All SMILE procedures were uneventful without any intraoperative or postoperative complications in any patient. SMILE induced a significant improvement in UDVA in both groups during six months follow-up period (*p* < 0.001 for both) (Table 1 and Figure 1).

Group 1 and group 2 had comparable mean CDVA preoperatively (−0.08 ± 0.08 logMAR versus –0.08 ± 0.08, resp.; *p* = 0.658) (Table 1, Figure 1(a)). However, at the first week after the operation, eyes in group 2 lost significantly more lines compared to the eyes in group 1, translating into better visual acuity in group 1 than group 2. The mean CDVA returned to the preoperative levels in both groups at postoperative month 1 (Table 1, Figure 1(a)). The mean UDVA was 0.07 ± 0.07 logMAR at day 1 and −0.08 ± 0.07 logMAR at month 6 after SMILE in group 1; and 0.29 ± 0.09 logMAR at day 1 and −0.06 ± 0.06 at month 6 after SMILE in group 2 (Table 1). The mean UDVA was significantly better in group 1 than group 2 in all postoperative assessment points from day 1 to month 6 (Table 1, Figure 1(b)).

### 3.3. Corneal Higher-Order Aberrations

**Figure 2:**
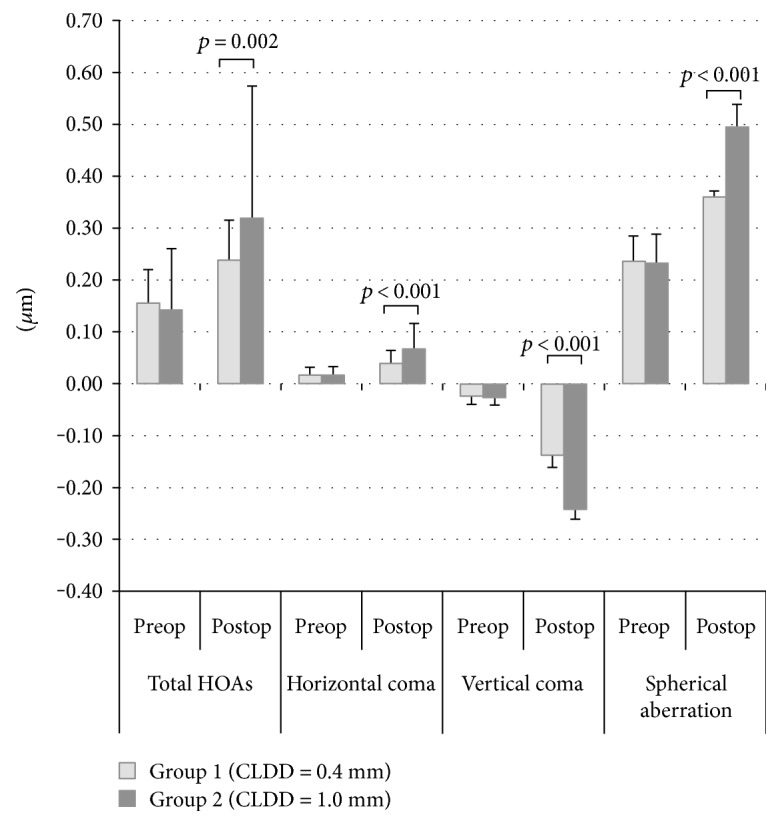
The mean higher-order aberrations (*μ*m) of group 1 and group 2 before (preop) and six months (postop) after SMILE procedure. Although study groups had similar aberrations preoperatively, group 1 was associated with significantly higher induction of total HOAs, horizontal coma, vertical coma, and spherical aberrations than group 2 after SMILE procedure. HOAs: higher-order aberrations.

The total HOAs, horizontal coma, vertical coma, and spherical aberrations increased significantly after SMILE in both groups (*p* < 0.001 for all, Table 1). The mean HOAs of group 1 and group 2 before SMILE procedure was 0.16 ± 0.06 *μ*m and 0.14 ± 0.12 *μ*m (*p* = 0.248, Table 1, Figure 2). There was also no significant difference between study groups in the horizontal coma, vertical coma, and spherical aberrations preoperatively (Table 1). However, HOAs was significantly higher in group 2 than group 1 postoperatively (0.32 ± 0.26 *μ*m and 0.24 ± 0.08 *μ*m, resp.; *p* = 0.002; Table 1, Figure 2). In other words, group 1 was associated with significantly less induction of total HOAs, horizontal coma, vertical coma, and spherical aberrations than group 2 after SMILE procedure (Table 1, Figure 2).

## 4. Discussion

In this prospective comparative study, we primarily showed that CLDD has an impact on the visual acuity in the early postoperative period after SMILE procedure. Narrow CLDD has a benefit of better visual outcome and higher induction of HOAs in SMILE during early recovery period.

SMILE has similar efficacy and safety profile with femtosecond LASIK with the advantage of lack of flap creation and associated risks of LASIK technique [7–10, 20]. However, longer recovery period of SMILE compared to LASIK is a well-known disadvantage in clinical practice, which surpasses its benefits [11]. According to the studies, the main weakness of SMILE has been a tendency toward a slight delay in visual recovery within the first week compared to other refractive surgeries [12, 14, 21]. Kamiya et al. found a tendency for a slight delay in UDVA recovery in the early postoperative period after SMILE [22]. It may take up to one month or more to achieve satisfactory uncorrected and corrected distance corrected visual acuity (UDVA and CDVA) after surgery [3]. This long recovery process associated with SMILE may be due to microirregularities on the corneal surface, prolonged surgical manipulations, or high interface reflectivity [3, 23]. Agca et al. found a difference in stromal response patterns between SMILE and LASIK operations in their study [23]. These different patterns effect the visual recovery. Due to this factor, they concluded to a possible delay in visual recovery of SMILE. When the stromal fibers are cut by the femtosecond laser, the interface distribution is modified. This results in a reduction in visual acuity in the first weeks of the postoperative period of SMILE [13, 24].

There has been no definite result with the various approaches suggested to overcome this problem, such as intraoperative cap repositioning, lowering laser energy, and changing cap thickness [15–18, 25]. Ji et al. reported that femtosecond energy levels effect the visual acuity [17]. To achieve better results in the early postoperative period, 115 nJ is recommended to be the maximum energy level. Also, Donate and Thaëron reported that energy level close to the plasma threshold during SMILE provides faster and better visual recovery [18].

In the present study, we evaluated the effect of CLDD on the visual outcome and HOAs in SMILE by comparing 0.4 mm versus 1.0 mm CLDD for the first time.

We found that regardless of CLDD, SMILE provided a statistically significant correction in sphere and spherical equivalent and UDVA without causing any significant change in CDVA at six months after the procedure. In clinical studies with up to 1 year postoperative follow-up, SMILE has been shown to be a reliable, efficient, and safe procedure for correction of high myopic refraction errors [22, 26, 27]. Therefore, our findings support the present literature data that SMILE effectively and safely corrects myopia and myopic astigmatism. Furthermore, we found that UDVA and CDVA were significantly better in group 1 than in group 2, indicating that CLDD 0.4 mm provided better visual acuity particularly during early postoperative period.

In excimer laser surgery, it is well known that higher myopic corrections induce more HOAs, which reduce the visual performance under reduced light causing night vision disturbances [28]. Therefore, an ideal excimer laser technique for correction of myopia should induce less HOAs and cause minimum loss or gain in CDVA.

Wavefront-guided LASIK results in less postoperative HOAs as compared to LASIK and femtosecond LASIK [29–31]. Also, SMILE has been reported to induce less HOAs and spherical aberrations than both LASIK and femtosecond LASIK [9, 32, 33]. In the comparison of SMILE and wavefront-guided LASIK, there are important procedural differences. There is no eye tracking and iris registration in SMILE and it relies on subjective fixation. In wavefront-guided LASIK operation, pupil shift is traced with iris registration which is an effecting factor in comparison of these two operations.

In the reports of Ye et al., it is cited that there is no difference in the induced total HOAs or SA between two operations [9]. In SMILE, there are more horizontal and vertical coma induced. Wavefront-guided LASIK is designed for compensating the induction of HOAs but as we have mentioned above, there is no iris registration in SMILE that compensates for pupillary cyclotorsion and offset to provide good centration. This centration is not as precise as in excimer laser eye tracker system [14]. In a report of Chen et al., the postoperative values are compared for SMILE and wavefront-guided LASIK and there was no substantial difference in trefoil, horizontal coma, spherical aberration, and total HOA of these operations [34]. The only difference is in vertical coma, and its increase after SMILE operation is due to the decentration of lenticule along the vertical axis. For minimizing the induced coma, it is essential to have an accurate centration in SMILE.

In the present study, we compared the anterior corneal aberration after 0.4 mm versus 1.0 mm CLDD in SMILE procedure and found that 0.4 mm CLDD results in less induction of total HOAs, horizontal coma, vertical coma, and spherical aberrations than 1.0 mm CLDD. This shows that the centration of SMILE procedure is probably more precise with 0.4 mm CLDD.

The main limitation of this study was the limited sample size, which precludes us reaching a definitive conclusion on the impact of CLDD on the visual acuity in the early postoperative period after SMILE procedure. Nevertheless, this study provides first evidence on the importance of CLDD in the clinical practice of SMILE. Further large-scale, randomized studies are needed to confirm our findings.

In conclusion, in comparison to standard CLDD of 1.0 mm, CLDD 0.4 mm is associated with better visual outcome and less induction of HOAs in SMILE in the short term. Therefore, narrow CLDD should be considered in SMILE to increase the visual acuity particularly in the early postoperative period.

## Figures and Tables

**Table 1:** Demographic and ocular findings of the study groups throughout the study.

		Group 1 (CLDD = 0.4 mm)	Group 2 (CLDD = 1.0 mm)	*p* value^a^
Number of patients		54	78	
Number of eyes		108	156	
Age (years), mean ± SD (range)		29.25 ± 5.36 (19–39)	29.54 ± 5.03 (20–40)	0.326
Gender (male/female)		33/21	46/32

Sphere (D)	Preoperative	−3.01 ± 1.81	−3.13 ± 1.72	0.582
	Month 6	−0.02 ± 0.36	−0.01 ± 0.42	0.943
	*p* value^b^	<0.001	<0.001	

Spherical equivalent (D)	Preoperative	−3.47 ± 1.78	−3.55 ± 1.80	0.750
	Month 6	−0.20 ± 0.37	−0.24 ± 0.43	0.412
	*p* value^b^	<0.001	<0.001	

CDVA (logMAR)	Preoperative	−0.08 ± 0.08	−0.08 ± 0.08	0.658
	Day 1	−0.07 ± 0.07	0.04 ± 0.07	<0.001
	Week 1	−0.07 ± 0.07	−0.04 ± 0.07	0.001
	Month 1	−0.08 ± 0.07	−0.07 ± 0.07	0.333
	Month 3	−0.08 ± 0.07	−0.07 ± 0.07	0.445
	Month 6	−0.08 ± 0.07	−0.08 ± 0.07	0.456
	*p* value^c^	0.089	<0.001	

UDVA (logMAR)	Day 1	0.07 ± 0.07	0.29 ± 0.09	<0.001
	Week 1	0.02 ± 0.06	0.11 ± 0.08	<0.001
	Month 1	−0.05 ± 0.07	0.02 ± 0.06	<0.001
	Month 3	−0.07 ± 0.07	−0.04 ± 0.06	<0.001
	Month 6	−0.08 ± 0.07	−0.06 ± 0.06	0.038
	*p* value^c^	<0.001	<0.001	

Total HOAs (*μ*m)	Preoperative	0.16 ± 0.06	0.14 ± 0.12	0.248
	Month 6	0.24 ± 0.08	0.32 ± 0.26	0.002
	*p* value^b^	<0.001	<0.001	

Horizontal coma (*μ*m)	Preoperative	0.02 ± 0.02	0.02 ± 0.02	0.937
	Month 6	0.04 ± 0.03	0.07 ± 0.05	<0.001
	*p* value^b^	<0.001	<0.001	

Vertical coma (*μ*m)	Preoperative	−0.02 ± 0.02	−0.03 ± 0.02	0.326
	Month 6	−0.14 ± 0.02	−0.24 ± 0.02	<0.001
		<0.001	<0.001	

Spherical aberration (*μ*m)	Preoperative	0.23 ± 0.05	0.23 ± 0.06	0.786
	Month 6	0.36 ± 0.01	0.49 ± 0.04	<0.001
	*p* value^b^	<0.001	<0.001	

CLDD: cap-lenticule diameter difference; SD: standard deviation; CDVA: corrected distance visual acuity; UDVA: uncorrected distance visual acuity; HOA: higher-order aberrations. ^a^Independent sample *t*-test for the difference between study groups. ^b^Paired *t*-test for the significance of intragroup change during study. ^c^Repeated measures of ANOVA for the significance of intragroup change during study.
